# Safeguarding cancer research funding by European charities amidst the COVID‐19 pandemic

**DOI:** 10.1002/1878-0261.12839

**Published:** 2020-11-22

**Authors:** Ioannis Tsagakis, Maria Papatriantafyllou

**Affiliations:** ^1^ Molecular Oncology Editorial Office Heidelberg Germany

**Keywords:** cancer charities, cancer research, COVID‐19, funding, pandemic, policy

## Abstract

The COVID‐19 outbreak has affected cancer research and cancer care. European cancer charities need to reconsider strategies for safeguarding income and supporting cancer researchers, in times when sustaining cancer research funding is more crucial than ever.

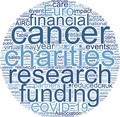

**Abbreviations**
AECC: Asociación Española Contra el Cáncer (Spanish Association Against Cancer)
AIRC: Associazione Italiana Ricerca Sul Cancro (Italian Foundation for Cancer Research)
COVID‐19: coronavirus disease 2019
CRUK: Cancer Research UK
ERC: European Research Council
EU: European Union

## 1 Introduction

The COVID‐19 pandemic is having severe consequences for cancer patient care [1, 2, 3, 4, 5]. Cancer patient screening and referrals are being delayed, ongoing clinical trials are being brought to a halt, and new ones are not being approved. Guidelines and clinical recommendations for cancer patients have already been laid out [1, 3, 5]. However, the cancer‐related effect of the pandemic is not restricted to patient care alone, but has spread to cancer research funding – the two aspects being inextricably linked.

National research budgets across Europe are threatened by cuts following the economic damage caused by the pandemic. European Union (EU) leaders budget of Horizon Europe — the research and innovation framework of the EU for the period of 2021–2027 — down to 84.9 billion Euro, a significant reduction compared to the original 94.4 billion Euro proposal put forward by the commission in May [6, 7, 8]. The European Research Council (ERC), the funding body that promotes excellence in research (including high‐risk high‐reward cancer research), will also be largely affected by such cuts, with potential long‐term consequences for cancer research and cancer treatment [9].

But not all funding for cancer research comes from governments and the EU [10]. In Europe, cancer charities provide funds for cancer research, in addition to their roles in supporting patients more directly. So, how have charities that support cancer research funding been affected? What challenges are they currently being faced with, and what strategies will help them to survive within a pandemic‐disrupted cancer research – cancer care ecosystem? We contacted European cancer charities to collect data and perspectives on the pandemic‐associated economic blow sustained so far, with a special focus on consequences for cancer research funding. Whilst several organizations are still gathering data and planning their next steps, this article summarizes emerging evidence on the impact of the COVID‐19 pandemic on European cancer charities. In addition, we highlight the need for innovative approaches and reciprocal action to maintain a high standard of cancer research.

## 2 Charities fuel cancer research

Cancer charities contribute to cancer care through direct patient support [11], but their indirect contribution through cancer research funding is equally crucial. European cancer charities provide financial aid for cancer research and clinical trial testing, as well as advocate for research with government and inform on policy. The specific contributions of cancer charities to research funding are diversified across Europe. Cancer charities are the backbone of cancer research funding in some Northern European countries, including the Scandinavic countries and the UK. By contrast, in Western Europe, financial support towards cancer researchers by charities is seen as complementary to national and European funding — although equally crucial, as it creates space for demand‐driven research funding. Charitable cancer research funding remains to be developed in Eastern Europe [12].

In all cases, however, cancer research funding by cancer charities often enables breakthroughs in cancer patient care by backing high‐risk, high‐reward research not otherwise funded. Cancer charity investments are also being used to indirectly foster collaborations between scientists, for instance, through the establishment of infrastructure for multidisciplinary research or acquisition of cutting‐edge technology. Scientific progress through cancer research might not be visible to cancer patients until results materialize in the form of a new treatment or new guidelines. Again, this is where cancer charities can bridge such disconnect by curating discussion forums, blog posts, and online information pages, to highlight the significance of advances in cancer research. These online platforms also provide accurate cancer care guidance and tie in information from various external resources, including national health services.

## 3 The financial toll of the COVID‐19 pandemic for European cancer charities

A major route via which charities generate income is fundraising. In response to the COVID‐19 pandemic in 2020, most European governments introduced self‐isolation and social distancing measures in early March. Accordingly, European cancer charities had to cancel or postpone fundraising events such as marathons (e.g. Race for Life and Relay‐for‐Life), walks (e.g. Kiltwalk and March for Men), flower sales, door‐to‐door fundraising, coffee mornings and concerts. This has translated in severe losses for charities that heavily relied on the spring and summer fundraising events (Table 1). Indicatively, the 2020 revenues from races, festivals, dinner galas and concerts were reduced by 10 million Euro for the Spanish Association Against Cancer (AECC).

**Table 1:** Expected income loss per European cancer charity in 2020/21. N/A, data not available; P.C., personal communication.

Cancer charity	Projected income loss (in Euro)	Loss as % of annual income	References
Cancer research UK (CRUK)	165 m	25%	[11]
Cancer Society of Finland	None	None	P.C.
Dutch Cancer Society	10–99 m	N/A	P.C.
French Cancer League	10 m	5–10%	P.C.
Italian Foundation for Cancer Research (AIRC)	20–25 m	20–25%	P.C.
Spanish Association Against Cancer (AECC)	20 m	23%	P.C.

Safety measures also translated into reduced retail activity of charity shops since they had to shut down, further exacerbating their income shortfall. Most charities rely on volunteers to keep events and retail shops up and running. The pandemic safety measures considerably reduced the number of available volunteers, the impact of which could echo into the coming years.

Events that ultimately materialized earlier in the year in a virtual format have had reduced success. For instance, the Norwegian Cancer Society reported that already in March they replaced their door‐to‐door fundraising event ‘Krafttak mot kreft’ with a virtual event, which was albeit met with limited success, ending up to 1.7 million Euro short in profit. ‘Many people are also facing their own financial uncertainty and may be less able to give money to charity’ notes Dr Rosie Loftus, Joint Chief Medical Officer at Macmillan Cancer Support, a UK‐based charity directly supporting cancer patients. Echoing this remark, the AECC disclosed a 2 million Euro shortfall in revenues from private donors. Corporate support has equally been affected: ‘most of our corporate partners have deviated their corporate social responsibility budgets to support COVID‐19 emergency programs and initiatives. This has decreased our revenues from corporate support by 2–3 million Euro', report AECC's president, Dr Ramon Reyes, and CEO, Noema Paniagua.

Back‐up plans that some cancer charities relied on for economic reinforcement of their budgets, such as assets placed in financial investments, have in several cases also fallen through. ‘Unfortunately, the financial market is currently very volatile, and the value of shares has plummeted. We expect a decrease in our budgeted financial income by 2.8–4.6 million Euro if the unrest in the financial markets does not subside’ explains N.M Compaoré, Norwegian Cancer Society's Special Advisor of International Affairs.

## 4 Implications for cancer research funding

In April, Cancer Research UK (CRUK), the UK's biggest contributor to cancer research funding, announced an estimated reduction in future research grants in the order of 20% [11]. This announcement came with additional corollaries for grant applicants since spring funding rounds were postponed to autumn, increasing competition for available funding. This unprecedented funding cut followed an expected combined income loss of 25% of the CRUK annual budget (~ £150 million, 165 million Euro), attributable to the impact of COVID‐19 on fundraising events and retail activity [1, 13]. In July, the projected annual income loss was reported at 30% (~ 160 million GBPs, 177 million Euro), with a further loss of 300 million GBPs expected in the next three years [14]. The latter is bad timing especially for the UK, as by the end of the Brexit transition period, rights to apply for EU funding, including ERC grants, may be relinquished, and hampered collaborations may further spark talent loss [15, 16]. Of note, the UK has so far ranked first among ERC funding beneficiaries with over 1600 researchers securing ERC funding in 2007–2013, compared to ~ 1000 beneficiaries in Germany or France [17].

The COVID‐19 financial toll has been felt by cancer researchers all around Europe, as several European cancer charities have announced delays in planned calls for projects or are faced with income deficits (Table 1). Initial estimations from the Dutch Cancer Society report an income loss in the order of tens of millions of Euros. Early estimates from the French Cancer League indicate a setback in the order of 5–10% of their income, a gap which may widen by the end of the year. Equally, the Norwegian Cancer Society has so far experienced losses of 12–13% of their annual income. Moreover, the Italian Foundation for Cancer Research (AIRC) and the AECC reported income shortfalls of 20–25% in the 2020 budget. Charities are also concerned about the long‐term impact of the pandemic on their income. Indicatively, the AECC predicts a loss of 5 million Euro in their expected 2021 revenues, merely as a consequence of the earlier 3‐month collapse in new member donor acquisition.

The AECC notes that a budget of 17 million Euro has been invested in cancer research this year, which is smaller compared to previous years as a consequence of delays or postponements of funding calls. Efforts to circumvent this issue include the launch of new research funding initiatives, such as the AECC Innova, reopening of some calls for projects, and introduction of programmes to sustain talent support and strengthen clinical research. In addition to experiencing delays in issuing funding to projects, French Cancer League’s cancer research budget could be cut by as much as 20%. Whilst the AIRC can afford to dig ‘deeper’ into their reserve funding to avoid research‐funding cuts in 2020–2021, their funding capacity may be affected later on, as according to Professor Federico Caligaris‐Cappio, Scientific Director of AIRC, ‘… from 2022 onward [it] will depend upon several different and so far unpredictable variables and scenarios’.

## 5 Resilient charities: the other side of the coin

However, not all European cancer charities have yet had to issue funding cuts. For example, the Cancer Society of Finland has not yet reported any income loss, despite facing a fiercer competition for fundraising. Similarly, Fondation ARC in France have still not felt the impact of the pandemic on their income. In contrast to other European cancer charities, Fondation ARC has maintained all planned calls for projects in 2020, confirmed no cuts to funding budgets and is facilitating grant extensions for projects that have secured funding. Furthermore, they launched a ‘Cancer & COVID‐19’ call for projects in April, with the scope of expanding research into the interaction between cancer and other pathologies. They have also announced a ‘rescue’ budget to ensure the financial security of PhD students and postdoctoral researchers who recently started projects, but have suffered delays due to the pandemic.

The Swedish Cancer Society appears equally immune to the pandemic financial strain at the moment. As key fundraising events were scheduled for autumn, the Swedish Cancer Society was not severely affected by early lockdown measures. This meant that there was sufficient time to act swiftly with asset relocation, as well as to adapt and include corporate partners in their financial strategies, which helped minimize financial shortfalls. It is worth noting that Sweden did not experience a strict lockdown in spring; therefore, retail activity might have been less affected in Sweden, compared to other European countries. As such, the Swedish Cancer Charity announced a slight stimulus in cancer research funding for this academic year, with the prospect of a further increase the following year. ‘The Swedish Cancer Society realizes that this is a critical time during which cancer research has to be fostered, even if this would imply taking some risks’ stated Ulrika Årehed Kågström, Secretary General, Swedish Cancer Society.

Nevertheless, it is important to remember that the pandemic is ongoing and lockdown rules of varying severity could extend into the next 18–24 months in the absence of a vaccine [18]. Equally, several European cancer charities find that it is still early to accurately assess the pandemic toll. This uncertainty perplexes the broader European landscape that is currently perceived as hostile for cancer researchers in need of financial support [7, 8]. Thus, cancer research funding needs to be safeguarded for the foreseeable future, and perhaps European‐wide initiatives need to be considered. Cancer researchers and cancer patients depend on European cancer charities and policymakers putting strategies in place in anticipation of a long economic dry spell.

## 6 Innovation as a means to weather the pandemic storm

‘Innovation and creativity have never been so crucial [for charities]’, notes Dr Rosie Loftus, Joint Chief Medical Officer at Macmillan Cancer Support.

Faced with cancellations and postponements of fundraising events, cancer charities had to devise internal strategies to mitigate income loss, adapt to the new norm of the pandemic lifestyle and as such modify their fundraising efforts. Several cancer charities have had to convert their fundraising efforts to fully virtual or hybrid formats (Table 2).

**Table 2:** Examples of virtual fundraising events from European cancer charities.

Cancer charity	Virtual fundraising event
Cancer Research UK	Marathon race
Dutch Cancer Society	TV broadcast show
Fondation ARC, France	Triathlon
German Cancer Aid	Marathon race
AIRC	Flower sales
Macmillan Cancer Support	Coffee morning
	Games Night In
	Game Heroes
Norwegian Cancer Society	Pink ribbon sales
	Marathon race
Spanish Association Against Cancer (AECC)	Marathon race
	Online streaming concert
Swedish Cancer Society	Pink ribbon sales

A structural reorganization including recruitment of personnel with digital competence helped to address the new needs of the Swedish Cancer Society in the current pandemic climate. ‘As digitalization requires investment, and setting up new working modalities is largely challenging, we need to regularly take a step back and decide what the new core functions should be, as well as monitor the situation closely’ notes Ulrika Årehed Kågström. Moreover, she indicated that the phrasing of campaigns had to be adapted to the new reality, as it is more crucial now than ever to communicate notions with caution. ‘The message of urgency must be delivered in a delicate manner, not necessarily connecting to the pandemic’, she adds.

Along the same spirit, Macmillan Cancer Support launched Games Night In, raising over 388, 000 GBPs/428,  000 Euro since April. The Dutch Cancer Society is planning to broadcast a fundraising campaign through national TV called ‘The Netherlands will stand up to Cancer’ in an attempt to raise donations. Fondation ARC rescheduled most of its fundraising activity to the end of the year as virtual meetings, including ‘Journées Jeunes Chercheurs’ (‘Young Researcher days’) and the Griffuel Prize ceremony. As lockdown regulations were eased in France in September, the Triathlon des Roses, a sports event dedicated to women with breast cancer, materialized following the latest safety guidelines.

Moreover, the French Cancer League is gearing up active marketing campaigns to extend their fundraising call throughout the year as well as planning to hold virtual sports events. The Norwegian Cancer Society has started organizing their annual ‘Pink Ribbon campaign’, which aims to generate donations based on pin sales and contributions of business partners in the name of breast cancer. Likewise, the Swedish Cancer Society involved corporate partners in their online retail activity relating to their pink ribbon campaign. Nevertheless, in the current COVID‐19‐inflicted financial crisis, it is expected that cancer research charity partners may be forced to restrict their contributions. However, there could be other ways in which a mutually beneficial collaboration could be achieved between the two sectors.

‘We envision a flexible approach towards businesses with which we have partnership agreements…’ says N.M. Compaoré, Norwegian Cancer Society.

## 7 Fighting cancer together: building alliances across sectors and borders

It is of paramount importance for cancer charities to diversify their fundraising initiatives and develop innovative strategies to secure a steady revenue. However, extraordinary circumstances require extraordinary measures, namely that cancer charities liaise with other cancer charities as well as partners in industry and governmental structures in pursuit of their mission in this new landscape. The Nordic Cancer Union, a collaborative body promoting cooperation among the Danish, Swedish, Finnish, Icelandic, Norwegian and Faroese cancer societies, sets a good example of how cancer charities could team up in times of crisis. In addition, the Swedish Cancer Society has plans to uphold cancer care by supporting patient organizations, which have been largely affected, mainly due to their smaller size.

Ulrika Årehed Kågström highlighted that collaborations among European cancer charities could be part of the solution: ‘We can work more closely together to share examples of successful new paths. We also have to remain outspoken about failures and losses, as even a single stroke in cancer research funding in one of the European countries will affect future cancer patients globally’. Along the same lines, Ramon Reyes and Noema Paniagua note: ‘we strongly believe it’s important to share best practices and a common work approach, as this isn’t an one runner challenge’. Flexibility seems to be the new keyword in this rapidly evolving pandemic situation, and flexible collaborations could be the way forward for cancer charities, which could focus on new ways to incentivize and liaise with industry.

Foundations or third‐party organizations from the corporate sector could constitute key partners to cancer charities for mutual economic benefit and pandemic‐related expenditure mitigation. Various regional trusts, retail businesses, lotteries, banks and other foundations in the UK have stepped up and offered millions of pounds for charities affected by the virus [19, 20], and cancer charities would be eligible for some of these funds.

Some governments are also offering support to cancer charities, especially in countries that rely more on charity‐sponsored cancer research. As part of a financial injection, the British government has devoted 750 million GBPs/825 million Euro to help charities and hospices across the country following the aftermath of COVID‐19 [21]. The British government also matched public donations made at the national TV broadcast event, BBC Big Night In, bringing the total amount fundraised by this event to 77 million Euro [21]. However, not all charities will be eligible for these governmental funds, according to CRUK [22]. To further support the supporters, the UK government has relieved some costs from charities by deferring VAT bills, exempting charity shops from business tax for a year and covering a significant proportion of charity staff salaries for those no longer working their regular hours [21].

This economic injection, although generous, is hardly adequate to account for immediate shortfall in funding created by the pandemic, let alone the longer‐term effects. To put the figure of the financial rescue package announced by the UK government into perspective, the projected income loss due to COVID‐19 was estimated to be close to 13 billion Euro according to the Chartered Institute of Fundraising and Charity Finance Group [23]. Thus, CRUK has partnered together with the UK Association of Medical Research Charities to request 341 million Euro from the government, specific to medical charities, as a fiscal ‘rescue’ package to narrow their revenue deficit in 2020/21 [24]. Cancer medical charities are also campaigning for Gift Aid tax relief to be increased from 20% to 33% [25].

Whilst no governmental rescue packages aimed specifically aimed cancer charities have been announced across Europe, governments in Italy and Norway are compensating cancer charities by financially supporting staff unable to work remotely. As in the UK, the Norwegian government is now providing reduced taxation for cancer charities. The French Cancer League and Cancer Society of Finland believe that offering a reduced tax rate when taxpayers donate to cancer charities could incentivize people to contribute towards combating the impact of COVID‐19 on charity funding. Likewise, the 5 × 1000 scheme in Italy has enabled AIRC to receive 60 million Euro in 2019, in the form of donations equal to 0.5% of taxpayer annual payments. Although contributions from this scheme normally reach charities every two years, the waiting time was reduced to one year given current conditions.

Campaigning and government lobbying are becoming increasingly decisive so that cancer charities can attract monetary attention from European governments and foundations. The Norwegian Cancer Society has been quite vocal in requesting support from the Norwegian Ministry of Health and Care Services, the Directorate of Health and various sustenance schemes from foundations, with limited success. Likewise, the Dutch Cancer Society is in the process of securing financial compensation for their budget from the government.

The European Commission can have a key role in supporting an endagered and fragmented cancer research funding landscape, especially in times of crisis. Already in 2010, the European Commission launched and co‐financed a European network of cancer funding organisations, TRANSCAN. The TRANSCAN network involves ministries, funding agencies and charities from about 20 countries across Europe. Continuation of this project within Horizon Europe is expected to ensure best use of the available (and occasionally reduced) cancer research budgets of charities and other organisations at the continental level, while promoting international researcher collaborations.

While the pandemic threatens sustainability of cancer research funding by charities, in the long run it may accelerate international collaborations, as well as facilitate actions targeting policy makers and the general public. The COVID‐19 crisis has brought research and health care in the limelight, forcing the public and politicians to realize the core value, complexity and timeframe of basic and translational research. This will boost the potential of national and intersectional concerted cancer plans and, hopefully, assist policies strategically formulated to fight a longer‐lasting epidemic such as cancer.
